# The Impact of Antioxidants on Vitiligo and Melasma: A Scoping Review and Meta-Analysis

**DOI:** 10.3390/antiox12122082

**Published:** 2023-12-06

**Authors:** Reinhart Speeckaert, Vedrana Bulat, Marijn M. Speeckaert, Nanja van Geel

**Affiliations:** 1 Department of Dermatology, Ghent University Hospital, Corneel Heymanslaan 10, 9000 Ghent, Belgium; 2 Department of Dermatology, University Hospital Centre Zagreb, 10000 Zagreb, Croatia; 3 Department of Nephrology, Ghent University Hospital, 9000 Ghent, Belgium

**Keywords:** vitiligo, melasma, antioxidant, oxidative stress

## Abstract

Reactive oxygen species (ROS) generated during melanogenesis make melanocytes particularly vulnerable to oxidative stress, influencing their survival and melanin synthesis. Oxidative stress, significantly present in vitiligo and recently also detected in melasma, triggers inflammatory cascades and melanogenesis, making antioxidants a promising therapeutic avenue. A systematic search was conducted on Embase and Pubmed to study the efficacy of antioxidants for treating vitiligo and/or melasma. Meta-analysis was performed to assess the difference in Melasma Severity Index (MASI) scores between baseline and follow-up. Various antioxidants like polypodium leucotomos, ginkgo biloba, catalase/superoxide dismutase, and vitamin E have potential in vitiligo. For melasma, vitamin C, silymarin, and niacinamide were among those showing promise in reducing pigmentation, with vitamin C displaying significant effects in meta-analysis. Different antioxidants improve both vitiligo and melasma, with an increased minimal erythema dose (MED) following UV exposure being significant for vitiligo and tyrosinase inhibition being crucial for melasma. However, the efficacy of individual antioxidants varies, and their exact mechanisms, especially in stimulating melanocyte proliferation and anti-inflammatory pathways, require further investigation to understand better and optimize their use.

## 1. Introduction

Reactive oxygen species (ROS) are part of the normal cellular metabolism produced by mitochondria, perixosomes, and melanosomes [1]. ROS are increased during inflammation and cancer but also due to environmental factors such as UV exposure. Oxidative stress deregulates the normal homeostasis of melanocytes, influencing melanogenesis and their survival [2]. The reactive oxygen species (ROS) released during melanogenesis render melanocytes particularly vulnerable to oxidative stress. Oxidation reactions, superoxide anion (O^2−^), and hydrogen peroxide (H_2_O_2_) are produced during melanin synthesis. Melanogenesis takes place in specialized organelles called melanosomes, which protects the other cellular components from oxidative damage [3]. The levels of H_2_O_2_ after UV exposure correlate inversely with skin pigmentation [4]. In contrast, strongly pigmented melanocytes are more susceptible to UVA-induced DNA damage then pigment cells with low concentrations of melanin [5]. This suggests that the oxidative damage due to the same environmental triggers is likely different across different skin phototypes.

Increased melanocyte cell death due to oxidative stress may contribute to the loss of melanocytes in vitiligo. Numerous studies have confirmed increased erythrocyte superoxide dismutase (SOD), superoxide dismutase in the skin, and decreased skin catalase levels in vitiligo patients [6]. Therefore, an elevated oxidative/antioxidative balance is clearly present in vitiligo. Even in non-lesional vitiligo skin, elevated SOD, glutathione peroxidase (GPx), and deregulated catalase have been documented. GPx converts hydrogen peroxide into water and oxygen. This suggests that oxidative stress is likely a contributor to the pathogenesis of vitiligo rather than a secondary phenomenon [7,8]. Oxidative stress can trigger a variety of inflammatory cascades. The misfolding of proteins due to oxidative stress initiates the unfolded protein response (UPR). The UPR induces the production of chemokines [e.g., Chemokine (C-X-C motif) ligand CXCL16] and dendritic cell activity, contributing to autoantigen presentation [9]. Additionally, the inflammatory environment due to the immune-mediated attack of melanocytes results in a further increase of free radicals and oxidative particles, creating a vicious loop of inflammation and oxidative stress.

More recently, increased oxidative stress has also been shown in melasma. Serum levels of malondialdehyde (MDA), an end product of lipid peroxidation, are increased in melasma patients and correlate with disease severity and disease extent [10]. Vitamin C was decreased in melasma. Serum SOD and glutathione were also reported to be increased in melasma, although no correlation with disease severity or extent was found [11].

Nonetheless, vitiligo and melasma have a clearly different pathogenesis. Vitiligo is primarily an immune-mediated disease where the destruction of melanocytes by IFN-γ producing cytotoxic T cells is considered the primary cause of the loss of melanocytes [12]. However, the inflammatory cascade is complex, involving multiple deregulated pathways. Oxidative stress is likely an initiating and driving factor of the inflammatory response and an epiphenomenon resulting from the production of chemokines and cytokines [13]. In contrast, melasma is caused by a focal hypermelanogenesis triggered through multiple factors, including a genetic predisposition, sun exposure, hormonal stimuli, increased vascularity, and skin inflammation. ROS are produced through external factors such as UV-exposure and during melanogenesis. Oxidative stress in melasma represents the multifactorial damage of melanocytes as deficient antioxidative mechanisms [6].

Both vitiligo and melasma are challenging skin disorders requiring long-term treatment. Antioxidants represent an attractive therapeutic option due to the very low risk of adverse events. In this review, we summarize the evidence around the use of antioxidants as a treatment for vitiligo and melasma and explore the similarities and differences in the types of antioxidants that are useful for both pigmentary disorders.

## 2. Materials and Methods

**Figure 1:**
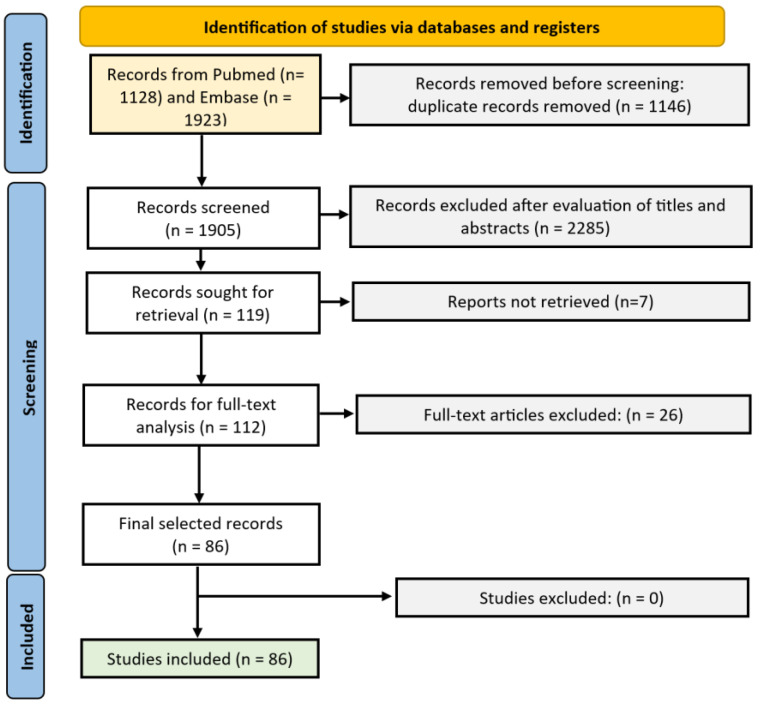
PRISMA flow diagram.

A systematic search was conducted in Embase and Pubmed from inception to July 2023. All articles investigating antioxidants as a treatment for vitiligo or melasma were included. First, a preliminary search was done to identify the most common antioxidants used for melasma and vitiligo to be included in the search query. As such, studies that used antioxidants (e.g., vitamin C) for vitiligo or melasma but did not mention the word ‘antioxidant’ or other search terms referring to oxidative stress could be identified. The final search strategy is added as supplementary material (Supplementary Data S1). The systematic search was done by 2 independent investigators, RS and MS. Only articles in English were considered. All types of original articles were taken into account, including letters and abstracts. Overlapping studies were excluded based on the author lists and descriptions of the same patient population. Only trials that included more than 5 patients were considered. Reviews were excluded. Data on the type of study design, patient size, and efficacy were extracted (Supplementary Data S2). For the vitiligo part, the study population was limited to vitiligo (non-segmental) patients. Patients with segmental vitiligo were excluded from the analysis. Both repigmentation and disease stabilization were considered relevant endpoints to assess the efficacy of antioxidative treatment. For melasma, the melasma severity index (MASI) or modified MASI (mMASI) were collected, if available. This review followed the PRISMA guidance and was not registered in PROSPERO. The PRISMA flow chart can be found in Figure 1. Studies on melasma using combination treatments were not selected if more than one antioxidant was different compared to the control group, as the individual contribution of each antioxidant is impossible to evaluate in these trials. Studies of antioxidants used in peelings were excluded.

Meta-analysis was not possible for vitiligo as the reported outcome measures were too heterogeneous, preventing statistical analysis. In contrast, a meta-analysis was done for antioxidants against melasma in case >2 studies that were available and reported a difference (±standard deviation) between baseline and follow-up MASI scores in the interventional and control groups. Funnel plots were created to check for bias. The significance level was set at *p* < 0.05.

## 3. Results

### 3.1. Results in Vitiligo (Table 1)

Thirty-three articles were found, including thirteen on catalase/superoxide dismutase, five on polypodium leucotomos, four on vitamin E, five on vitamin B12, three on ginkgo biloba, and three on lipoic acid.

#### 3.1.1. Catalase/Superoxide Dismutase

Nine studies compared the use of (pseudo)catalase/superoxide dismutase to another treatment or placebo. In 6/9 publications, phototherapy was concurrently used. Moreover, 4/9 found significantly higher repigmentation rates for (pseudo)catalase/superoxide dismutase compared to the control group. In contrast, 4/9 reported no difference compared to the placebo group or patients without treatment. However, one study did find comparable repigmentation of topical catalase/superoxide dismutase compared to 0.05% betamethasone (18.5 ± 93.14% with betamethasone and 12.4% ± 59 with C/DSO) [14]. Two studies were performed with oral treatment of which one resulted in a significant positive effect compared to placebo [15]. The second study did not include a comparison group but mentioned excellent repigmentation in 90% of patients on the face and the dorsum of the hands [16].

#### 3.1.2. Polypodium Leucotomos

Five studies used oral polypodium leucotomos, of which four included a control group [17,18,19,20]. Moreover, 3/4 of the studies resulted in improved repigmentation rates with polypodium leucotomos, whereas the only report not mentioning an improved outcome included only eight patients (comparison *n* = 4 vs. *n* = 4). In all four trials, a combination with phototherapy was used.

#### 3.1.3. Vitamin E

Three out of four studies using oral vitamin E reported more repigmentation than placebo or no treatment. However, in two of the positive studies, the vitamin supplements included other antioxidants such as α-lipoic acid, vitamin C, carotenoids, and fruit extracts [21,22]. A small study of 15 treated and 15 non-treated patients found no difference in using a multivitamin and antioxidant supplement [23]. For now, we can only state that the addition of vitamin E to supplements containing other antioxidants might lead to increased repigmentation.

#### 3.1.4. Vitamin B12

Five studies were carried out with vitamin B12, although only two had a comparison group. Neither study found a significant difference in oral vitamin B12 compared to no treatment [24,25]. Three studies without a control group reported beneficial results with significant repigmentation in at least half of the patients and a stop of progression from 64–100% [26,27,28]. However, the data are insufficient to confirm an added value of vitamin B12 in vitiligo.

#### 3.1.5. Ginkgo Biloba

Three studies with oral ginkgo biloba were published. Parsad et al., 2003 [29] found a significantly higher cessation of spread with ginkgo biloba compared to placebo. Another study reported a significant decrease in serum IL-6 in vitiligo patients receiving ginkgo biloba for 4 weeks compared to placebo [30]. Szczurko et al., 2011 found a significant decrease in disease progression and disease extent (mean repigmentation of 15%) 3 months after initiation of ginkgo biloba in young vitiligo patients (12–35 years) [31].

#### 3.1.6. α-Lipoic Acid

α-lipoic acid has been investigated in 3 randomized controlled trials. Two studies used oral α-lipoic acid in monotherapy but resulted in non-significant results. However, in the study of Li et al., 2015 repigmentation ≥ 75% was seen more frequently (55%) compared to the control group (37%) [32]. Another study used a combination of different antioxidants, including α-lipoic acid, vitamin C, vitamin E, and other ingredients, which gave significantly more repigmentation compared to placebo after 24 weeks [21]. Nonetheless, the sample size was small (14 treated vs. 11 placebo patients). The efficacy of α-lipoic acid in vitiligo remains, therefore, unconfirmed.

#### 3.1.7. Other

Other antioxidants have a limited number of studies. In patients receiving phototherapy, oral selenium induced follicular repigmentation in 60.9% of patients, whereas this was only 29.1% in the placebo group [33]. However, another report found no difference in oral selenium used in combination with other antioxidants compared to no antioxidants with phototherapy [23]. Turmeric cream resulted in more repigmentation compared to placebo in an RCT of 4 months [34].

**Table 1:** Antioxidants and vitiligo.

Antioxidant	(P)lacebo/No Treatment; (O)ther; (N)o Comparison	(T)opical or (O) Oral	Author	UV	Study Design	(B)etween/(W)ithin	# Pts	Weeks	Outcome	
**Catalase/superoxide dismutase (SOD)**										
Oral gliadin-protected SOD	(P) Placebo	O	Fontas et al., 2021 [15]	Y	RCT	B	25/25	24	**↑**	I: improvement VES:19.9% (SE:4.6); VES30:6 (24%); VES50:4 (16%) C: improvement VES:8.8% (SE 4.7); VES30:3 (12%); VES50:1 (4%)
Pseudocatalase	(P) Nothing	T	Schallreuter et al., 2008 [35]	Y	Uncontrolled, retrospect.	B	71/10	32–52 w	**↑**	I: repigmentation: 100%: 28/71 (39.4%); >75%: 38/71 (53.2); 0%: 5/71 (7%); C: repigmentation: 50%: 1 (face); none in all other
Pseudocatalase	(P) Placebo	T	Schallreuter et al., 2002 [36]	Y	Random., prosp.	B	39/20	3 w	**↑**	I: Significantly more follicular repigmentation in the face compared to controls; 100% cessation of spread
SOD, copper, zinc, vit.B12, calcium pantothenate	(P) Nothing	T	Soliman et al., 2014 [37]	Y	Open label, prosp.	W	30	12 w	**↑**	I: response excellent = 20%; good = 26.7%; moderate = 22.2%; poor = 22.2%; none = 8.9%. C: response: 48.9% moderate; 42.2% poor; 8.9% none.
Vitix gel	(P) Nothing	T	Yuksel et al., 2009 [38]	Y	Open	B	15/15	24 w	**↗**	I: Repigmentation: *n* = 1 (>75%); *n* = 4 (51–75%); *n* = 10 (26–50%); *n* = 6 (≤25%) C: Repigmentation: *n* = 0 (>75%); *n* = 2 (51–75%); *n* = 8 (26–50%); *n* = 11 (≤25%)
Pseudocatalase/SOD gel (+ tacrolimus)	(P) Nothing (+tacrolimus)	T	Alshijab et al., 2020 [39]	N	RCT	B	25/24	36 w	**=**	Percentages of pigmentation on 3, 6, and 9 months: group 1= 23.9%, 40.4%, and 60% vs. group 2 = 23.2%, 40.7%, and 62.4%.
Pseudocatalase	(P) Placebo	T	Bakis et al., 2009 [40]	Y	RCT	B	14/18	24 w	**=**	N.S.
Pseudocatalase/SOD	(P) Placebo	T	Naini et al., 2012 [41]	N	RCT	W	23	6 m	**=**	N.S.

Catalase/SOD	(O) 0.05% betamethason	T	Sanclemente et al., 2008 [14]	N	RCT	B	25	40 w	**↘**	I: repigmentation: 12.4% ± 59 C: repigmentation: 18.5% ± 93

Catalase and SOD	(N)	T	Kostović et al., 2007 [42]	Y	Prosp., 1 arm	N	19	6 m	/	Repigmentation: >50%: 11/19 (57.9%); >75%: 3/19 (15.79%); 26–50%: 6/19 (31.58%); 1–25%: 1/19 (5.26%); none: 1/19 (5.26%); No new lesions
Pseudocatalase	(N)	T	Patel et al., 2002 [43]	Y	Prosp., 1 arm	N	32	24 w	/	No obvious improvement; 10/26 with at least some improvement of either the hands or face
Pseudocatalase and calcium	(N)	O	Schallreuter et al., 1995 [16]	Y	Prosp. 1-arm	N	33	36 m	/	Excellent repigmentation of the face and dorsum of the hands in 90%. Focal vitiligo: 90–100% repigmentation in all cases; No new lesions
Cucumis melo extract	(N)	T	Schallreuter et al., 2011 [44]	N	Retrosp., one-arm	N	53	1–24 w	/	No repigmentation without phototherapy Repigmentation in 3/9 with 311 nm phototherapy
**Polypodium leucotomos (PLE)**										
PLE	Placebo	O	Pacifico et al., 2021 [17]	Y	RCT, assessor-blinded	B	23/21	6 m	**↑**	I: repigmentation: head/neck: excellent = 85%; trunk: moderate-excellent = 92%; extremities: moderate = 83%; C: repigmentation: head/neck: excellent = 25%; trunk: moderate-excellent = 44%; extremities: moderate = 12%;
PLE	Placebo	O	Reyes et al., 2005 [18]	Y	RCT	B	10/9	12 w	**↑**	I: >50% repigmentation significantly higher compared to placebo
PLE	Placebo	O	Middelkamp et al., 2007 [19]	Y	RCT	B	25/24	26 w	**↗**	I: trend towards more repigmentation compared with the placebo group
PLE	Placebo	O	Salazar et al., 2013 [20]	Y	RCT	B	4/4	24 w	↘	I: depigmentation: Baseline: 35 ± 21%; FU: 16 ± 17%. C: depigmentation: Baseline: 26 ± 13%; FU: 6 ± 4%.

PLE	/	O	Mohammad et al., 1989 [45]	N	Prosp. 1-arm	/	22	5 m	/	100% cured of the disease. This successful treatment coincided with the hottest months of the year.
**Vitamin E**										
Phyllanthus emblica fruit extracts, vitamin E, carotenoids	Nothing	O	Colucci et al., 2014 [22]	Y	Random	B	65/65	24	**↑**	I: significant mild repigmentation in the head and neck (*p* = 0.019) and on the trunk (*p* = 0.051), a higher but not significant repigmentation for each body site, and higher stable disease (*p* = 0.065).
a-lipoic acid, vit C, vit E, polyunsat. fatty acids, cysteine	Placebo	O	Dell’Anna et al., 2007 [21]	Y	RCT	B	14/11	24 w	**↑**	I: repigmentation: >75% in 8/17(47%; *p* < 0.05 vs. placebo);0–75%; 4/17 (23.5) C: repigmentation: >50% in 2/11(18%)
Vitamin E	Nothing	O	Elgoweini et al., 2009 [46]	Y	Prosp. Random	B	12/12	6 m	**↑**	I: Marked to excellent repigmentation in 8/11 (72.7%); No new lesions C: Marked to excellent repigmentation in 5/9 (55.6%); No new lesions
Vit E, beta-carotene, vit C, selenium, copper, zinc, manganese	Nothing	O	Jayanth et al., 2002 [23]	Y	Random., not blinded	B	15/15	12	**=**	N.S.
**Vitamin B12**										
2 groups (1) vit D and (2) vit D and vit B12	Nothing	O	Iraji et al., 2017 [24]	Y	Open randomized	IB	20/20/20	24 w	**?**	N.S
Vit B12 and folic acid	Nothing	O	Tijoe et al., 2002 [25]	Y	Prosp., open	B	14/3	1 y	**=**	No significant difference in repigmentation at any time point.
**Vitamin C**										
Vit C, vit B12, folic acid	/	O	Don et al., 2006 [27]	Y	Prosp, 1-arm	/	9	10 m	/	Significant repigmentation in all patients. Stop disease progression in 9/9
Vit B12 and folic acid	/	O	Juhlin et al., 1997 [26]	Y	Prosp, 1-arm	/	100		/	Repigmentation in 52/100, Stop of disease progression in 64%
Vit C, vit B12	/	O	Sendrasoa et al., 2019 [28]	N	Prosp, 1-arm	/	308	3–18 m	/	>76% repigmentation in 50 (65.7%)
**Ginkgo biloba**										
Ginkgo biloba	Placebo	O	Parsad et al., 2003 [29]	N	RCT	B	25/22	6 m	**↑**	I: Marked to complete repigmentation in 10 patients C: Marked to complete repigmentation in 2 patients Significant cessation of spread G. biloba (*p* = 0.006): 20/25 vs. 8/22
Ginkgo biloba	Placebo	O	Abu-Raghif et al., 2013 [30]	Y	RCT., single blinded	B	12/12	8 w	**↗**	I: VASI baseline: 6.42 ± 4.08; VASI FU: 6.17 ± 4.27; Difference: −0.25 C: VASI baseline: 3.75 ± 2.81; VASI FU: 3.88 ± 2.77; Difference: +0.13

Ginkgo biloba	/	O	Szczurko et al., 2011 [31]	N	Prosp. open-label	/	11	12 w	/	Mean percent improvement: 15%. Significant impact on arresting the spread of vitiligo: total spreading score from 2.7 to −1.1 (*p* ≤ 0.001)
**α-lipoic acid**										
α-lipoic acid	Placebo	O	Li et al., 2015 [32]	Y	RCT	B	26/24	6 m	**↗**	I: repigmentation ≥75% in 55%; 51–75% in 35% C: repigmentation ≥75% in 36.84%; 51–75% in 47.37%.
α-lipoic acid	Placebo	O	Sun et al., 2020 [47]	Y	RCT	B	37/28	6 m	**↘**	I: >50% repigmentation: 37.8% (14/37) C: >50% repigmentation: 42.9% (12/28)
**Other**										
Selenium	/	O	Tsiskarishvili et al., 2016 [33]	Y	?	B	17/18	5 w	**?**	I: follicular repigmentation: 60.9%; C: follicular repigmentation: 29.1%
Turmeric	Placebo	T	Jalalmanesh et al., 2022 [34]	N	RCT	B	24	4 m	**↑**	I: significant reduction in the size of the lesions following applying turmeric cream compared to placebo (independent-sample *t*-test, mean ± SE: 29.31 ± 5.31 for drug vs. −21.36 ± 11.00 for placebo, *p* < 0.05).

↑ (dark green) Significantly increased efficacy compared to the control group; ↗ (light green) increased without statistical significance or statistical significance of the difference between the final follow-up and baseline not mentioned; = (grey) no difference with the control group; ↘ (brown) decreased efficacy compared to the control group without statistical significance or statistical significance of the difference between the final follow-up and baseline not mentioned; ↓ (red) significantly decreased efficacy compared to the control group; ? the statistical significance is unclear; / no comparison possible because lack of control group

### 3.2. Melasma (Table 2; Supplementary Table S1)

Fifty-three articles were found on melasma, including eighteen on vitamin C, six on niacinamide, six on cysteamine, five on azelaic acid, four on silymarin, three on polypodium leucotomos, two on tomato extract/lycopene, two on zinc sulfate and seven on other antioxidants.

#### 3.2.1. Vitamin C

A total of 20 articles described the use of topical vitamin C for melasma. Nine reports performed a comparison with placebo or no treatment, although none of these studies used vitamin C in monotherapy. This is remarkable as other antioxidants have been investigated in monotherapy for melasma. This makes it difficult to determine the exact efficacy of vitamin C. Seven out of nine studies found a significantly increased improvement of the MASI with vitamin C compared to the control group. Nine studies investigated the efficacy of vitamin C compared to another treatment. A slightly lower improvement of the MASI was observed with topical vitamin C compared to topical tranexamic acid after dermapen microneedling, although the difference remained not significant [48]. Transdermal injections with either vitamin C or tranexamic acid resulted in similar results with slightly higher improvement in the tranexamic acid group, although again without reaching statistical significance [49]. Vitamin C iontophoresis outperformed with a 42% improvement in MASI glycolic acid 70% peels, which exhibited only a 22% improvement [50]. Overall, vitamin C has a reproducible efficacy on melasma, although other agents, such as tranexamic acid, may be more potent.

**Figure 2:**
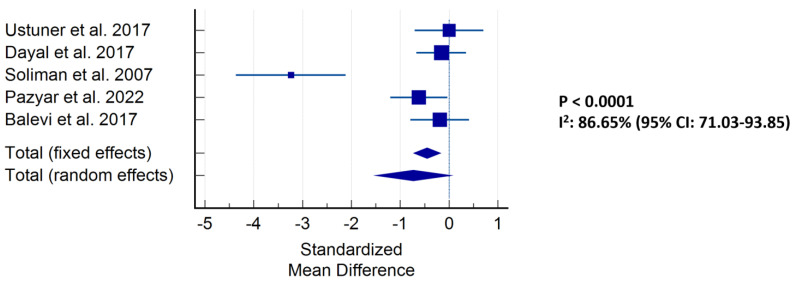
Meta-analysis of studies investigating vitamin C against melasma for all studies that reported a change in MASI from baseline to follow-up in the interventional and placebo groups [51,52,53,54,55].

Meta-analysis resulted in a highly significant (*p* < 0.0001) result pointing to a benefit of vitamin C against melasma compared to placebo or no treatment (Figure 2). Egger’s test and Begg’s test showed no signs of publication bias. A funnel plot identified the study of Soliman et al., 2007 as an outlier, although the meta-analysis without inclusion of the report remained significant (*p* < 0.001) [51].

#### 3.2.2. Niacinamide

The findings concerning the skin-brightening capacity of niacinamide have been mixed. A split-face study in 21 patients documented a 1.38–2.08 fold improvement compared to placebo [56]. In this trial, niacinamide was formulated into cationic liposomes to facilitate percutaneous absorption. This result was confirmed in another split-face trial, showing a decreased melasma area and improvement in hyperpigmentation [57]. Other reports also mentioned a reduction in melasma, although significance was not always reached. Comparison of niacinamide with hydroquinone, triple combination, and intradermal tranexamic acid resulted in a slightly higher average MASI decrease of 70% with hydroquinone compared to 62% for niacinamide and no significant difference compared to the triple combination or intradermal tranexamic acid [58,59]. The findings for niacinamide are, therefore, promising.

#### 3.2.3. Cysteamine

Compared to placebo, topical cysteamine 5% resulted in two trials with a significantly higher decrease in MASI score after 4 months of treatment [60,61]. Moreover, 5% cysteamine cream induced a 38% decrease in the mMASI after 120 days. This was less compared to 4% hydroquinone (−53%), although no significant difference was observed between the two treatments [62]. Sepaskhah et al., in 2022, reported similar non-significant findings when comparing topical 5% cysteamine with hydroquinone 4% and ascorbic acid 3%. Again, the mMASI decreased most in the hydroquinone group without significant difference compared to 5% cysteamine, comparable to another small RCT [63,64]. Finally, a similar improvement was observed with 5% cysteamine compared to tranexamic acid and mesotherapy [65].

#### 3.2.4. Silymarin

Topical sylimarin 0.1% and 0.2% clearly outperformed placebo. Comparison of sylimarin with hydroquinone or low fluence 1064 Q Switched Nd:YAG laser was done in three studies. In none of these reports, silymarin performed significantly worse or better than the comparator treatment [66,67,68]. The comparable efficacy of silymarin to hydroquinone could place this antioxidant firmly into the therapeutic arsenal for melasma.

#### 3.2.5. Polypodium Leucotomos

Oral polypodium leucotomos in combination with 4% hydroquinone led to a mMASI decrease of 54.9% after 12 weeks, significantly less than monotherapy with hydroquinone (44.4% decrease) [69]. A 28.8% improvement was found after 12 weeks of using polypodium leucotomos without any additional treatment, which was non-significantly better than placebo with a 13.8% skin brightening [70]. Another similar study only found improvement in the polypodium leucotomos-treated group, while the placebo group worsened [71]. Based on the current evidence, a modest efficacy of polypodium leucotomos in melasma seems plausible.

#### 3.2.6. Azelaic Acid

Five trials were done to compare topical azelaic acid to other depigmentation treatments. Overall, 20% azelaic acid performed slightly worse compared to hydroquinone 4%, tranexamic acid 10%, diclofenac gel 2.3%, and topical tranexamic acid 3%, despite one trial reporting a better efficacy of azelaic acid compared to hydroquinone 4% when combined with tranexamic acid [72,73,74,75,76]. These results point to a confirmed efficacy, although other agents may be more potent.

#### 3.2.7. Tomato/Lycopene

Oral tomato extract lead to more improvement in patients already using topical hydroquinone, with a 3-point decrease in mMASI compared to a 1.2 mMASI decrease without this supplement [77]. Topical tomato lycopene 0.05% and wheat bran extract 3.45% resulted in a modest but significant difference compared to placebo [78].

#### 3.2.8. Zinc Sulfate

Topical zinc sulfate performed significantly worse than hydroquinone 4% in two trials. More than double the MASI reduction was seen with hydroquinone at 4% compared to zinc sulfate at 10% solution [79,80].

#### 3.2.9. Other

Many other antioxidants have been tested in melasma with variable outcomes, although confirmatory studies are lacking. Mulberry extract oil, glutathione, oral pycnegol, and rucinol serum scored significantly better than placebo or no treatment [62,81,82,83,84,85]. Petroselinum Crispum was non-inferior compared to 4% hydroquinone, although limited improvement was seen in both groups after 8 weeks [85].

### 3.3. Effects of Antioxidants (Figure 3, Supplementary Table S2)

Remarkably, all described antioxidants for melasma and vitiligo have evidence of a tyrosinase-inhibiting effect. Reduction of melanin/melanosome transfer from melanocytes to keratinocytes is only present for two antioxidants, niacinamide, whose efficacy has only been reported in melasma, and vitamin E, which can be useful for both melasma and vitiligo. In contrast to what might be expected, no antioxidants for vitiligo have evidence for melanocyte proliferation or migration. Vitamin E and catalase/superoxide dismutase decrease melanocyte proliferation. Most antioxidants for melasma exert no effect on melanocyte proliferation. All reported antioxidants carry an anti-inflammatory capacity and/or reduce chemokine production. A reduction of the minimal erythema dose (MED) does not seem to be the primary mechanism of antioxidants against melasma. In contrast, all antioxidants effective against vitiligo reduce the MED. Overall, it seems that the inhibition of tyrosinase is crucial for melasma, whereas the reduction of inflammation, the decreased chemokine production, and lower inflammation following UV exposure (MED) are more characteristic of an effective antioxidant against vitiligo.

**Table 2:** Antioxidants and melasma.

Antioxidant	(P = Placebo/Nothing; O = Other Treatment)	(T)opical/(O)ral	Author	Study Design	(B)etween/(W)ithin	# Pts	Duration	Outcome	
**Vitamin C**									
4% liquiritin mixed in 5% ascorbic acid	(P) 4% liquiritin	T	Akram et al., 2013 [86]	RCT	W	41/41	6 m	**↑**	I: improvement in MASI: *n* = 36 (88%) C: improvement in MASI: *n* = 25 (61%)
20% TCA Peel with 5% Ascorbic Acid	(P) 20% TCA peeling	T	Dayal et al., 2017 [53]	Unblinded, prosp., rand.	/	30/30	12 w	**↑**	I: Baseline MASI: 23.55 ± 4.62; 12 wk: 9.50 +/− 5.31 C: Baseline MASI:23.6 ± 4.08; 12 wk: 15.10 +/− 4.44
Vitamin C iontophoresis	(P) Distilled water iontophoresis	T	Huh et al., 2003 [87]	RCT	W	21	12 w	**↑**	Significant difference between the ¢L value of the vitamin C- and placebo-treated sites (*p* = 0.03).
Intradermal tranexamic acid + top. ascorbic acid	(P) Intradermal tranexamic acid + placebo	T	Pazyar et al., 2022 [54]	Split-face comparative	W	24	12 w	**↑**	I: Decrease in MASI: 2 points (baseline: 4.61 (SD 1.54); FU: 2.61 (SD 1.14) C: Decrease in MASI: 1.29 points (baseline: 4.49 (SD 1.48); FU: 3.20 (SD 1.21) Significantly lower final MASI in the intervention group.
TCA peel and ascorbic acid	(P) TCA peel	T	Soliman et al., 2007 [51]	Prop. trial (randomized?)	W	15/15	16 w	**↑**	I: Baseline MASI: 13.753 ± 4.101; FU: 7.73 ± 4.203; average decrease: 6.023 C: Baseline MASI: 15.413 ± 2.881; FU: 12.32 ± 3.381 average decrease: 3.093
Microneedling with vit C + Q-switched Nd:YAG	(P) Q-switched Nd:YAG	T	Ustuner et al., 2017 [52]	RCT	W	16	6 m	**↑**	I: MASI at baseline: 7.04 ± 4.55; final: 2.49 ± 2.30 C: MASI at baseline: 6.13 ± 4.94; final: 4.52 ± 3.49
Medlite C6 q-1064 laser+ vit C, E and ferulic acid	Medlite C6 q-1064 laser	T	Y et al., 2023 [88]	Ranomized split-face	W	61	14 w	**↑**	I: difference in MASI score: t = 17.25 C: difference in MASI score: t = 9.78
Salicylic acid peeling + vitamin C mesotherapy	(P) Salicylic acid peeling	T	Balevi et al., 2017 [55]	Single-blinded RCT	B	23/21	2 m	**↗**	I: baseline MASI:16.68 ± 11.57; FU: 5.32 ± 2.68; Decrease: 11.36 C: baseline MASI: 15.81 ± 10.51; FU: 13.97 ± 10.86; Decrease: 1.84. N.S.
1064-nm Q-switched Nd:YAG + ultrasonic vitamin C	(P) 1064-nm Q-switched Nd:YAG	T	Lee et al., 2015 [89]	Split-face prosp.	W	8	4 sess + 3 m	**↗**	I: VAS after the first treatment: 3.00 ± 0.53; final VAS: 1.37 ± 0.52 C: VAS after the first treatment: 3.75 ± 0.89; final VAS: 1.50 ± 0.53 Significant > VAS reduction with vit. C at different time points, not at the final FU

Vitamin C + microneedling	(O) PRP + microneedling	T	Abdel-Rahman et al., 2021 [90]	Prosp. split-face	W	10	6 sess + 1 m	**↑**	I: Baseline MASI: 11.75 ± 2.75; FU: 2.78 ± 0.67; MASI reduction: 76.29% C: Baseline MASI: 12.06 ± 2.39; FU: 5.86 ± 1.06; MASI reduction: 46.13%
Vitamin C iontophoresis	(O) glycolic acid 70% peel	T	Sobhi et al., 2012 [50]	Prosp., single blinded (?)	W	14	6 sess	**↑**	I: Baseline MASI: 8.3143 ± 2.815; FU: 4.778 ± 2.793; decrease: 3.535 C: Baseline MASI: 7.9714 ± 2.536; FU: 6.2143 ± 2.725; decrease: 1.757
Vitamin C after microneedling	(O) tranexamic acid after microneedling	T	El Attar. et al., 2022 [48]	Prosp., rand., uncont.	W	20	12 w	**↘**	I: baseline hemi-MASI: 0.90–21.60; FU: 0.60–18.0; decrease: 45.94% C: baseline hemi-MASI: 0.90–21.60; FU: 0.0–16.20; decrease: 53.76%.
Transdermal injections of vitamin C	(O) transdermal tranexamic acid	D	Zhao et al., 2020 [49]	RCT	W	17	2 m	**↘**	I: 6.94 ± 4.28; FU: 3.32 ± 3.30; Difference: 3.62 ± 2.79 C: 7.03 ± 3.84; FU: 2.97 ± 2.62; Difference: 4.06 ± 2.62
Vitamin C iontophoresis	(O) Multivitamin ionotophoresis	T	Choi et al., 2010 [91]	Split-face	W	20	12	**=**	Both groups reported equal improvement in subjective self-assessment and colorimetry
Vitamin C + microneedling	(O) Tranexamic acid + microneedling	T	Raza et al., 2020 [92]	Split-face prosp.	W	30	6 w	**=**	I: 13% excellent response, 43% good response, 30% fair improvement C: 16% excellent response, 40% good, 26% mild improvement
3% vitamin C derivative	(O) Plant extracts, including orchid	T	Tadokoro et al., 2010 [93]	Split-face, prosp. study	W	18	8 w	**=**	Both formulations significantly increased the average color value (lightness) of the pigmented spots (*p* < 0.01)
20% vitamin C solution + microneedling	(O) Tranexamic acid + microneedling	T	Tahoun et al., 2020 [94]	Split-face prosp.	W	30	16 w	**=**	I: MASI baseline: 6.34 ± 3.78; FU: 3.04 ± 2.64 C: MASI: baseline: 5.98 ± 3.58); FU: 3.64 ± 2.62
5% ascorbic acid	(O) 4% hydroquinone	T	Espinal-Perez et al., 2004 [95]	RCT, split-face	W	16	16 w	**↓**	I: Patients’ assessment: excellent: *n* = 2, good: *n* = 8, moderate: *n* = 4, mild: *n* = 2 C: Patients’ assessment: excellent: *n* = 8, good: *n* = 7, moderate: *n* = 1, mild: *n* = 0
**Niacinamide**									
Niacinamide formulated into cationic liposomes	(P) Control solution	T	Lee et al., 2022 [56]	Prospective, split-face	W	21	4 w	**↑**	I: 1.38–2.08-fold improvement compared to the control solution (*p* < 0.05)
Niacinamide 4%	(P) Placebo	T	Campuzano et al., 2019 [96]	RCT	B	10/10	8 w	**↗**	I: Baseline MASI: 15.4 ± 6.7; FU: 10.4 ± 5.1 C: Baseline MASI: 9.1 ± 1.4; FU: 7.1 ± 1.2.
Niacinamide + sunscreen	(P) Placebo + sunscreen	T	Hakozaki et al., 2002 [57]	RCT, split-face	W	18	8 w	**↗**	4–6 weeks: niacinamide + sunscreen showed significant increase in Lvalue (skin lightness) vs. placebo. No significance at 8 weeks (*p* = 0.059)
Niacinamide + sunscreen	(P) Placebo + sunscreen	T	Goh et al., 2012 [97]	RCT	B	30/30	84 d	**=**	MASI scores showed a significant reduction in patients treated with the study cream (6.0 to 4.6) and vehicle cream (6.6 to 4.7) (N.S.).

Niacinamide 4%	(O) Hydroquinone 4%	T	Navarrete et al., 2011 [59]	RCT, split-face	W	27	8 w	**↘**	I: Baseline MASI: 3.7 (95% CI, 2.9–4.4); FU: 1.4 (95% CI, 3.3–4.7) C: Baseline MASI: 4 (5% CI, 90.9–1.8); FU: 1.2 (95% IC, 0.8–1.6)
Niacinamide	(O) Kigman, intrad. tranexamic acid	T	Giasante et al., 2020 [58]	RCT	B	10/10	8 w	**=**	Triple combination, topical niacinamide, and intradermal tranexamic acid have similar responses.
**Cysteamine**									
Cysteamine 5%	(P) Placebo	T	Farshi et al., 2017 [60]	RCT	B	20/20	4 m	**↑**	I: MASI baseline: 18.1 ± 8.1; FU: 8.03 ± 5.2 C: MASI baseline: 13.2 ± 7.4; FU: 12.2 ± 7.4
Cysteamine 5%	(P) Placebo	T	Mansouri et al., 2014 [61]	RCT	B	25/25	4 m	**↑**	I: MASI baseline: 17.2 ± 8.1; MASI follow-up: 7.2 ± 5.5 C: MASI baseline: 13 ± 8.1; MASI follow-up: 11.6 ± 7.9

Cysteamine 5%	(O) tranexamic acid mesotherapy	T	Karrabi et al., 2022 [65]	Single-blind, rand.	B	27/27	2 m	**=**	I: mMASI baseline: 11.68 ± 2.70; FU: 6.32 ± 2.11 C: mMASI baseline: 10.43 ± 2.69; FU: 5.52 ± 2.55
Cysteamine 5%	(O) 4% hydroquinone	T	Lima et al., 2020 [62]	quasi-rand., evaluator-blinded	B	20/20	120 d	**=**	I: median (IQR): mMASI baseline: 9 (6–12); After: 5 (4–8) C: median (IQR): mMASI baseline: 6 (3–8); After: 2 (1–3) Mean reduction mMASI was 38% for CYS and 53% for HQ (*p* = 0.017).
Cysteamine 5%	(O) Hydroquinone 4% + vit C 3%	T	Sepaskhah et al., 2022 [63]	single-blind, RCT	B	31/34	4 m	**=**	I: decrease in mMASI from 6.69 ± 2.96 to 4.47 ± 2.16 C: decrease in mMASI from 6.26 ± 3.25 to 3.87 ± 2.00 in the HC group.
Cysteamine 5%	(O) Hydroquinone	T	Nguyen et al., 2021 [64]	Rand., double blinded trial	B	5/9	16 w	**↘**	I: reduction in mMASI: 1.52 ± 0.69 (21.3%) C: reduction in mMASI: 2.96 ± 1.15 (32%). N.S.
**Silymarin**									
Silymarin	(P) No treatment or placebo	T	Altaei et al., 2012 [98]	RCT	B	32/32	4 w	**↑**	I: (0.1%) MASI_start_: 17.1 ± 3.12; FU: 0; (0.2%); MASI_start_: 16.5 ± 2.8; FU: 0 C: baseline MASI: 16.8 ± 3.2; FU: 17 ± 3.4

Silymarin	(O) Low Fluence 1064 Nd:YAG	T	Ibrahim et al., 2021 [68]	RCT	B	25/25	3 m	**↗**	I: mMASI baseline: 9 (IQR: 5.4–12.1); mMASI FU: 2.3 (1.2–6.6) C: mMASI baseline: 7.3 (IQR: 4.8–10.3)); mMASI FU: 2.1 (1–4.7)
Silymarin	(O) Hydroquinone	T	Wattanakrai et al., 2022 [67]	RCT, split-face	W	23	3 m	**↗**	I: Modified MASI reduction: 17.97% C: Modified MASI reduction: 7.11%
Silymarin	(O) Hydroquinone	T	Nofal et al., 2019 [66]	Prosp. clinical trial	B	14/14/14	3 m	**↘**	I: (0.7%): baseline MASI: 18.56 ± 5.58; FU: 10.96 ± 4.48; difference: 39.21% (1.4%): baseline MASI: 21.75 ± 8.47; FU: 14.88 ± 7.79; difference: 33.84% C: baseline MASI: 16.64 ± 9.02; FU: 8.81 ± 5.68; difference: 46.75%
**Polypodium leucotomos**									
Polypodium leucotomos	(P) Placebo	O	Martin et al., 2012 [71]	RCT	B	21	12 w	**↑**	I: significantly improved mean (MASI) scores (5.7 to 3.3; *p* < 0.05), while the placebo group did not (4.7 to 5.7; *p* > 0.05).
Polypodium leucotomos + 4% hydroquinone	(P) Placebo + hydroquinone 4%	O	Goh et al., 2018 [69]	RCT	B	33	12 w	**↑**	I: baseline mMASI: 6.8; final: −54.9% C: baseline mMASI: 6; final: −44.4%
Polypodium leucotomos	(P) Placebo	O	Ahmed et al., 2013 [70]	RCT	B	16/17	12 w	**=**	The MASI scores similarly showed improvement in both groups without significant intergroup differences (*p* = 0.62).
**Azelaic acid**									
Azelaic acid 20% + oral tran. acid + sunscr.	(O) Hydroq 4% + oral tran. acid + sunscr.	T	Akl et al., 2021 [72]	RCT	B	25/25	4 m	**↗**	I: mMASI baseline: 17.06 (+/−1.51); mMASI FU: 5.58 (+/−1.28) C: mMASI baseline: 17.77 (+/−1.45); mMASI FU: 7.18 (+/−1.31)
20% azelaic acid	(O) 10% tranexamic acid cream	T	Das et al., 2020 [73]	RCT, split-face	B	16/16	12 w	**=**	Composite malar area pigment score (CMAPS) of tranexamic acid (1.73 ± 1.68) less than azelaic acid (2.08 ± 1.70). N.S.
20% azelaic acid cream	2.3% diclofenac gel	T	Arpornpattanapong et al. 2023 [76]	Single blind, split face	W	20	12	**↘**	The difference in mMASI between diclofenac and azelaic acid was insignificant (*p* = 067); patient satisfaction was significantly higher for diclofenac.
Azelaic acid 20%	(O) Hydroquinone 4%	T	Emad et al., 2013 [75]	Prosp, split-face	B	33	20 w	**↘**	I: baseline mMASI: 7.88 ± 3.27; FU mMASI: 3.47 ± 2.88; reduction: 55% C: baseline mMASI; 7.8 ± 3.36; FU mMASI: 3.11 ± 2.91; reduction: 61%
Oral tranexamic acid + topical 20% Azelaic Acid	(O) Oral + topical 3% tranexamic acid	T	Malik et al., 2019 [74]	Rand, prosp	B	50/50	6 m	**↘**	I: baseline MASI: 34 ± 13; FU: 10.62 ± 7.43 C: baseline MASI: 33.7 ± 12; FU: 6.06 ± 5.06
**Tomato/Lycopene**									
Tomato extract + hydroquinone 4%	(P) Hydroquinone 4%	O	Afriliana et al., 2020 [77]	Double-blind, rand.	B	31/31	12 w	**↑**	I: mMASI baseline: 5.25; after: 1.2; decrease: 3 C: mMASI baseline: 6.0; after: 4.2; decrease: 1.2
Lycopene 30 mg + 4% hydroquinone	(P) Placebo + 4% hydroquinone	T	Avianggi et al., 2022 [99]	Double-blind RCT	B	59	12 w	**↑**	The difference in MASI scores after therapy in the treatment group had a significant decrease compared to the control group.
**Zinc**									
10% Zinc Sulfate Solution	(O) Hydroquinone 4%	T	Iraji et al., 2012 [79]	RCT, investig.-blinded	B	36/36	6 m	**↓**	I: difference in MASI: 0.7 ± 0.7 C: difference in MASI: 2.7 ± 1.6. More significant reduction for HQ vs. zinc.
10% Zinc Sulfate Solution	(O) Hydroquinone 4%	T	Yousefi et al., 2014 [80]	RCT	/	40/42	2 m	**↓**	I: baseline MASI: 6.3 ± 2.1; FU MASI: 5.1 ± 2.0; decrease in MASI: 18.6% ± 20.8 C: baseline MASI: 6.4 ± 1.6; FU MASI: 3.9 ± 1.4; decrease in MASI: 43.5% ± 15.5
**Glutathione**									
Tranex. acid mesoth. + vit C + glutathione	(P) Tranex. acid mesotherapy + vit C	T	Iarji et al., 2019 [100]	RCT	W	30	12 w	**↑**	I: mMASI decrease: of 3.046 ± 1.25 (*p*-value < 0.001) C: mMASI decrease: 1.82 ± 0.88 (*p*-value < 0.001).
Glutathione + microneedling	(P) Microneedling	T	Mohamed et al., 2023 [83]	Split face, non-blinded	W	29	3 m	**↑**	I: baseline hemi-mMASI: 4.21 ±2.08; FU hemi-mMASI: 1.96 ± 1.30 C: baseline hemi-mMASI: 4.06 ± 1.91; FU hemi-mMASI: 2.31 ± 1.45
**Other**									
Mulberry extract oil	(P) Placebo	T	Alvin et al., 2011 [81]	RCT	B	25/25	8 w	**↑**	I: Baseline MASI: 4.076 ± 0.24; FU: 2.884 (±0.25); mean difference: 1.19 C: mean difference MASI: 0.06
Rucinol serum	(P) Placebo	T	Khemis et al., 2007 [84]	RCT, split-face	W	32	12 w	**↑**	I: clinical pigmentation score at baseline: 7.5 +/− 1.9; follow-up: 6.2 +/− 2.3 C: clinical pigmentation score at baseline: 7.5 +/− 1.9; follow-up: 6.7 +/− 2.1.
75 mg pycnogenol 2x/d	(P) Placebo	O	Lima et al., 2020 [82]	RCT	B	22/22	60 d	**↑**	I: mMASI_baseline_: 9.1 (4.1); FU: 4.6 (3.4); mMASI reduction: 4.4 (3.1) C: mMASI_baseline_: 9.2 (4.2); mMASI FU: 6.4 (4.3); mMASI reduction: 2.7 (2.5)
Petroselinum Crispum (Parsley)	(O) hydroquinone 4%	T	Khosravan et al., 2017 [85]	RCT	B	25/25	8 w	**=**	I: Baseline MASI: 6.66 ± 4.39; FU MASI: 4.92 ± 3.07 C: Baseline MASI: 6.68 ± 3.24; FU MASI: 5.06 ± 2.66. N.S.

↑ (dark green) Significantly increased efficacy compared to the control group; ↗ (light green) increased without statistical significance or statistical significance of the difference between the final follow-up and baseline not mentioned; = (grey) no difference with the control group; ↘ (brown) decreased efficacy compared to the control group without statistical significance or statistical significance of the difference between the final follow-up and baseline not mentioned; ↓ (red) significantly decreased efficacy compared to the control group; ? the statistical significance is unclear; / no comparison possible because lack of control group

## 4. Discussion

Beneficial results of a variety of antioxidants have been reported in both vitiligo and melasma. For vitiligo, most studies were performed with (pseudo)catalase or superoxide dismutase. Due to the mixed findings, definitive conclusions are difficult. SOD is inactivated via passage through the gastrointestinal tract. Oral gliadin-protected SOD may prevent the deactivation of this enzyme, possibly explaining the variability in efficacy [15]. Remarkably, no reports were found on (pseudo)catalase or superoxide dismutase in melasma despite a submitted study in clinicaltrials.gov with oral-gliadin-protected SOD, last updated in September 2021. Therefore, it is difficult to state whether the lack of published studies about (pseudo)catalase/SOD in melasma also represents a lack of clinical efficacy.

For polypodium leucotomos, multiple reports have been published both for vitiligo and melasma. In vitiligo, polypodium leucotomos has been confirmed to improve repigmentation in combination with UV exposure. It reduces acute inflammation following UV irradiation via inhibition of COX-2, increased removal of cyclobutane pyrimidine dimers, and prevention of oxidative DNA damage [101]. The reported dosages ranged from 480–720 mg/day. Polypodium leucotomos reduces the activation of Opsin-3 after exposure to blue light. Opsin-3 activates tyrosinase, leading to melanogenesis. A decreased cell death was also observed [102]. Schalka et al., 2014 showed not only an increased minimal erythema dose (up to 20.37%) with polypodium leucotomos but also an increased minimal pigmentary dose (17.41%), confirming its dual activity against vitiligo and melasma [103].

Ginkgo biloba appears to be a notable exception because its oral use has not (yet) been linked to an increase in MED. The topical application of ginkgo biloba exhibits a solar protective capacity in vitro [104]. Interestingly, 2/3 of the identified trials did not combine ginkgo biloba with phototherapy. Especially its potential capacity to decrease JAK2/STAT3 signaling is of interest [105]. Ginkgo biloba decreases serum inflammatory markers such as CRP, IL-6, and TNF-α [106]. Despite its capacity to inhibit α-MSH-induced melanogenesis, the evidence for melasma is scarce [107]. This is probably due the fact that ginkgo biloba is a weaker tyrosinase inhibitor than ascorbic acid [108].

Almost all antioxidants inhibit tyrosinase directly or indirectly. Vitamin C carries the highest evidence, with most melasma trials demonstrating a clear efficacy of topical treatment. Nonetheless, the effect seems less than tranexamic acid [48,49]. Vitamin C reduces tyrosinase in a dose-dependent manner, indicating that its efficacy may not be directly related to an antioxidative effect [109]. As all antioxidants reported to be beneficial for melasma inhibit tyrosinase, it is difficult to assess whether the strength of the antioxidative capacity plays a major role. Vitamin C has been used in two comparative trials of vitiligo, although both trials used combination supplements. One study showed beneficial results, whereas the second trial mentioned no significant repigmentation.

Multiple studies have used vitamin E both in vitiligo and melasma, showing positive results in general, although its efficacy is difficult to determine due to the different combination treatments used. Only one small study (2 groups of 12 patients) with vitamin E in monotherapy reported a higher degree of >75% repigmentation compared to no treatment [46]. Vitamin E has an indirect modulating capacity on the activation of T cells by reducing proinflammatory cytokines (TNF-α, IL-6…) and prostaglandin E2 [110].

For vitiligo, antioxidants appear to have a limited effect on the direct stimulation of melanocytes (e.g., melanocyte proliferation). Increasing the minimal erythema dose (MED) following UV exposure appears to be an important factor determining the efficacy of antioxidants in vitiligo. Most vitiligo studies have investigated antioxidants in clinical trials using concomitant UV exposure. Decreasing oxidative stress and associated inflammation may prevent the autoimmune destruction of newly developing melanocytes or migrating melanocytes after UV exposure. A similar mechanism was observed with other topical and systemic treatments for vitiligo with increased repigmentation rates, combining anti-inflammatory treatments with phototherapy. However, the standardization of outcome measures in vitiligo is needed to allow better comparability and meta-analyses.

It should be noted that the antioxidants used for pigmentary disorders have a very diverse nature. Some are enzymes (e.g., catalase, SOD), others are vitamins (Vit C, Vit E, niacinamide), thiolamines (e.g., cysteamine), flavonoids (e.g., sylimarin), carotenoids (e.g., lycopene), tripeptides (e.g., glutathione), fatty acids (e.g., α-lipoic acid) or saturated dicarboxylic acids (e.g., azelaic acid) [110,111,112,113,114]. Natural-derived antioxidants often contain a complex mixture of compounds. Examples are polypodium leucotomos (containing p-coumaric, ferulic, caffeic, vanillic, and chlorogenic acids) or ginkgo biloba (containing flavonoids and terpenoids) [101,106]. These compounds often have pleiotropic effects, and the different mechanisms contributing to the improvement of pigmentary disorders can be difficult to determine. Additionally, the physicochemical, biopharmaceutical, and pharmacokinetic properties can influence the results of clinical trials. Further research is therefore needed to elucidate the mechanisms through which these diverse antioxidants contribute to the treatment of pigmentary disorders and to optimize their delivery and efficacy in clinical settings.

## 5. Conclusions

In conclusion, antioxidants can be useful for both vitiligo and melasma. Although some agents have a combined effect against vitiligo and melasma, others seem to display a narrower mode of action.

## Figures and Tables

**Figure 3:**
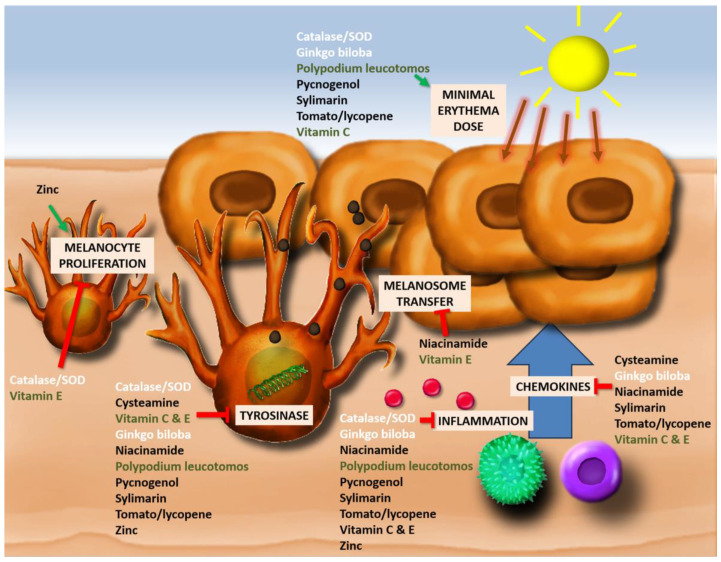
Impact of antioxidants on melanogenesis, melanosome transfer, melanocyte proliferation, inflammation, chemokine production, and minimal erythema dose (MED). (Green: antioxidants with efficacy for both vitiligo and melasma; white: antioxidants for vitiligo; black: antioxidants for melasma).

## Data Availability

Data are available upon request.

## Supplementary Materials

The following supporting information can be downloaded at: https://www.mdpi.com/article/10.3390/antiox12122082/s1, Supplementary Data S1: Search Strategy and Prisma Flow Diagram; Supplementary Data S2: PRISMA checklist; Supplementary Table S1: Studies about antioxidants in melasma without a control group; Supplementary Table S2: Influence of antioxidants on tyrosinase activity, melanosome transfer, melanocyte proliferation/differentiation and/or migration; Supplementary Table S3: Summary of the placebo-controlled trials using antioxidants for vitiligo and/or melasma [115,116,117,118,119,120,121,122,123,124,125,126,127,128,129,130,131,132,133,134,135,136,137,138,139,140,141,142,143,144,145,146,147,148,149,150,151,152,153,154,155,156,157,158,159,160,161,162,163,164,165,166,167].
