# Supplementary material for: The Impact of Antioxidants on Vitiligo and Melasma: A Scoping Review and Meta-Analysis
Source: Antioxidants (Basel). 2023 Dec 6;12(12):2082. doi: 10.3390/antiox12122082 (PMC10740621; doi:10.3390/antiox12122082)
Supplement: Supplementary file 1 [file antioxidants-12-02082-s001.zip › antioxidants-2715991-supplementary.pdf]

Supplementary Data S1: Search strategy

Date: from inception until October 20, 2023

Databases: Pubmed, Embase

Search strategy:

Vitiligo: Vitiligo AND oxida* OR oxidative stress OR antioxida* OR reactive oxygen species OR reactive oxygen OR catalase OR H2O2 OR superoxide OR selenium OR vitamin a OR vitamin c or ascorbic acid or homocystein or hydroxyproline or quercetin or vitamin B12 or folic acid or curcumin or flavonoid or baicalin or ginkgo biloba or galangin or pyrostegia venusta or pseudocatalase or polypodium leucotomos or cucumis or manganese or lipoic acid or carotenoids* or malondialdehyde or lipid peroxidation or comet or vitamin e or glucose-6-phosphate dehydrogenase or glutathione-6-transferase or niacinamide or cysteamine or silymarin or azelaic acid or tomato or lycopene or zinc or phytic acid or pycnogenol

Melasma: Melasma AND oxida* OR oxidative stress OR antioxida* OR reactive oxygen species OR reactive oxygen OR catalase OR H2O2 OR superoxide OR selenium OR vitamin a OR vitamin c or ascorbic acid or homocystein or hydroxyproline or quercetin or vitamin B12 or folic acid or curcumin or flavonoid or baicalin or ginkgo biloba or galangin or pyrostegia venusta or pseudocatalase or polypodium leucotomos or cucumis or manganese or lipoic acid or carotenoids* or malondialdehyde or lipid peroxidation or comet or vitamin e or glucose-6-phosphate dehydrogenase or glutathione-6-transferase or niacinamide or cysteamine or silymarin or azelaic acid or tomato or lycopene or zinc or phytic acid or pycnogenol

Supplementary Data S2: PRISMA checklist

Section and Topic	Item #	Checklist item	Location where item is reported
**TITLE**			
Title	1	Identify the report as a systematic review.	P1
**ABSTRACT**			
Abstract	2	See the PRISMA 2020 for Abstracts checklist.	P1
**INTRODUCTION**			
Rationale	3	Describe the rationale for the review in the context of existing knowledge.	P1-2
Objectives	4	Provide an explicit statement of the objective(s) or question(s) the review addresses.	P2-3
**METHODS**			
Eligibility criteria	5	Specify the inclusion and exclusion criteria for the review and how studies were grouped for the syntheses.	P2-3
Information sources	6	Specify all databases, registers, websites, organisations, reference lists and other sources searched or consulted to identify studies. Specify the date when each source was last searched or consulted.	P2-3, Supplementary Data S1
Search strategy	7	Present the full search strategies for all databases, registers, and websites, including any filters and limits used.	P2-3, Supplementary Data S1
Selection process	8	Specify the methods used to decide whether a study met the inclusion criteria of the review, including how many reviewers screened each record and each report retrieved, whether they worked independently, and if applicable, details of automation tools used in the process.	P2-3, Supplementary Data S1
Data collection process	9	Specify the methods used to collect data from reports, including how many reviewers collected data from each report, whether they worked independently, any processes for obtaining or confirming data from study investigators, and if applicable, details of automation tools used in the process.	P2-3, Supplementary Data S1
Data items	10a	List and define all outcomes for which data were sought. Specify whether all results that were compatible with each outcome domain in each study were sought (e.g. for all measures, time points, analyses), and if not, the methods used to decide which results to collect.	P2-3, Supplementary Data S1
	10b	List and define all other variables for which data were sought (e.g. participant and intervention characteristics, funding sources). Describe any assumptions made about any missing or unclear information.	P2-3, Supplementary Data S1
Study risk of bias assessment	11	Specify the methods used to assess risk of bias in the included studies, including details of the tool(s) used, how many reviewers assessed each study and whether they worked independently, and if applicable, details of automation tools used in the process.	N/A
Effect measures	12	Specify for each outcome the effect measure(s) (e.g. risk ratio, mean difference) used in the synthesis or presentation of results.	P2-3
Synthesis	13a	Describe the processes used to decide which studies were eligible for each synthesis (e.g. tabulating the study intervention characteristics	P2-3,

Section and Topic	Item #	Checklist item	Location where item is reported
methods		and comparing against the planned groups for each synthesis (item #5)).	Supplementary Data S1
	13b	Describe any methods required to prepare the data for presentation or synthesis, such as handling of missing summary statistics, or data conversions.	P2-3, Supplementary Data S1
	13c	Describe any methods used to tabulate or visually display results of individual studies and syntheses.	P2-3, Supplementary Data S1
	13d	Describe any methods used to synthesize results and provide a rationale for the choice(s). If meta-analysis was performed, describe the model(s), method(s) to identify the presence and extent of statistical heterogeneity, and software package(s) used.	P2-3, Supplementary Data S1
	13e	Describe any methods used to explore possible causes of heterogeneity among study results (e.g. subgroup analysis, meta-regression).	P2-3, Supplementary Data S1
	13f	Describe any sensitivity analyses conducted to assess robustness of the synthesized results.	N/A
Reporting bias assessment	14	Describe any methods used to assess risk of bias due to missing results in a synthesis (arising from reporting biases).	N/A
Certainty assessment	15	Describe any methods used to assess certainty (or confidence) in the body of evidence for an outcome.	P2-3, Supplementary Data S1
**RESULTS**			
Study selection	16a	Describe the results of the search and selection process, from the number of records identified in the search to the number of studies included in the review, ideally using a flow diagram.	P3, Figure 1
	16b	Cite studies that might appear to meet the inclusion criteria, but which were excluded, and explain why they were excluded.	P3
Study characteristics	17	Cite each included study and present its characteristics.	P3-17
Risk of bias in studies	18	Present assessments of risk of bias for each included study.	N/A
Results of individual studies	19	For all outcomes, present, for each study: (a) summary statistics for each group (where appropriate) and (b) an effect estimate and its precision (e.g. confidence/credible interval), ideally using structured tables or plots.	P3-17
Results of syntheses	20a	For each synthesis, briefly summarise the characteristics and risk of bias among contributing studies.	P3-17
	20b	Present results of all statistical syntheses conducted. If meta-analysis was done, present for each the summary estimate and its precision	P3-17

Section and Topic	Item #	Checklist item	Location where item is reported
		(e.g. confidence/credible interval) and measures of statistical heterogeneity. If comparing groups, describe the direction of the effect.	
	20c	Present results of all investigations of possible causes of heterogeneity among study results.	P3-17
	20d	Present results of all sensitivity analyses conducted to assess the robustness of the synthesized results.	N/A
Reporting biases	21	Present assessments of risk of bias due to missing results (arising from reporting biases) for each synthesis assessed.	N/A
Certainty of evidence	22	Present assessments of certainty (or confidence) in the body of evidence for each outcome assessed.	P3-17
**DISCUSSION**			
Discussion	23a	Provide a general interpretation of the results in the context of other evidence.	P18-19
	23b	Discuss any limitations of the evidence included in the review.	P18-19
	23c	Discuss any limitations of the review processes used.	P18-19
	23d	Discuss implications of the results for practice, policy, and future research.	P18-19
**OTHER INFORMATION**			
Registration and protocol	24a	Provide registration information for the review, including register name and registration number, or state that the review was not registered.	P2-3
	24b	Indicate where the review protocol can be accessed, or state that a protocol was not prepared.	P2-3
	24c	Describe and explain any amendments to information provided at registration or in the protocol.	N/A
Support	25	Describe sources of financial or non-financial support for the review, and the role of the funders or sponsors in the review.	P19
Competing interests	26	Declare any competing interests of review authors.	P19
Availability of data, code and other materials	27	Report which of the following are publicly available and where they can be found: template data collection forms; data extracted from included studies; data used for all analyses; analytic code; any other materials used in the review.	P19

From: Page MJ, McKenzie JE, Bossuyt PM, Boutron I, Hoffmann TC, Mulrow CD, et al. The PRISMA 2020 statement: an updated guideline for reporting systematic reviews. BMJ 2021;372:n71. doi: 10.1136/bmj.n71

For more information, visit: http://www.prisma-statement.org/

Supplementary Table S1: Studies about antioxidants in melasma without a control group.

Antioxidant	Compared to (P = placebo/ nothing; O= other treatment)	(T)opical or (O) Oral	Author	Study design	Inter- or intra*	# pts	Duration	Outcome : Significantly (↑) better or (↓) worse compared to the control or other treatment; Not significantly better (↗) or worse (↘) or equal (=) compared to control or other treatment or unclear (?)
**Vitamin C**								
**25% L-Ascorbic Acid**	No control	T	Hwang et al. 2009 [115]	Open-label trial	/	40	16 w	MASI baseline: 15.60; week 16: 12.03, p < .0001
**Microneedling with topical vitamin C**	No control	T	Ismail et al. 2019 [116]	Single arm prosp.	/	30	12 w	Baseline MASI: s 8.61 ± 4.45; Final MASI: 5.75 ± 4.16 in the last session (P < 0.0001).
**30% tetrahexyldecyl (THD) ascorbate serum + mineral sunscreen**	No control	T	Kelm et al. 2020 [117]	Single arm, prosp.	/	10	12w	All subjects showed an improvement in hyperpigmentation with an average improvement of 33.7%.
**vitamin C-iontophoresis**	No control	T	Yoo et al. 2001 [118]	Single arm, prosp.	/	15	6w	Decreased MASI and light reflectance were noted at the end of 6 weeks. Significant clinical improvement in the melasma was seen compared to before treatment.
**Pycnogenol**								
**Pycnogenol 100 mg**	No control	O	Campos et al. 2014 [119]	Single arm trial	/	29	2 m	Not effective (11.11% - 3/27), moderately effective (37.03% - 10/27), effective (48.14% - 13/27), and very effective (3.7% -1/27)
**Pycnogenol French maritime pine (Pinus pinaster)**	No control	O	Ni et al. 2002 [120]	Single arm trial	/	30	30 d	The average pigmentary intensity of the 30 women decreased significantly (p < 0.001).
**Other**								
**Cysteamine 5%**	No control	T	Hsu et al. 2012 [121]	Single arm, prospective	/	30	6 w	Cysteamine cream showed a considerable efficacy in the treatment of epidermal melasma
**Beta-carotene**	No control	T	Kar et al. 2002 [122]	Single arm prosp.	/	31	24 w	All nine cases (2 grade-II and 7 grade III) showed further clearing of the pigmentation to the next lower grade. In conclusion, topical application of b-carotene lotion appears to be effective and safe for melasma.
**Peeling with Amino Acid Filaggrin Based Antioxidants (AFAs)**	No control	T	Park et al. 2003 [123]	Single arm prosp.	/	14		AFAs peeling is an effective treatment modality in Korea and several repeelings improve the therapeutic effect
**Zinc Sulfate 10% Solution**	No comparison	T	Sharquie et al. 2008 [124]	Single arm prosp.	/	14	3 m	The mean MASI score before treatment was 9.45, which changed to 4.70 after therapy. This corresponds to a percentage improvement of 49.78% and was statistically significant (p = 0005).
**Red gingseng**	No control	O	Song et al. 2011 [125]	One-arm, prospective	/	25	24w	Baseline MASI: 8.76±6.56; FU: 5.49±3.93

**Proanthocyanidin-Rich Extract from Grape**	No control	O	Yamakoshi et al. 2004 [126]	Single arm prosp.	/	12	11 m	First 6 months: slightly improved chloasma in 10 of the 12 women (83%, $p < 0.01$) and following 5 months of intake improved or slightly improved chloasma in 6 of the 11 candidates (54%, $p < 0.01$).
**Ellagic acid**	No control	T	Yokoyama et al. 2001 [127]	Single arm prosp.	/	15	?	73.3% effective (11 cases/15 cases) for chloasma

Supplementary Table S2: Influence of antioxidants on tyrosinase activity, melanosome transfer, melanocyte proliferation/differentiation and/or migration

	Inhibition of tyrosinase activity	Inhibition of melanin/ melanosome transfer	Stimulation of melanocyte proliferation/differentiation Melanocyte migration	Inhibition of inflammation	Inhibition of chemokines	MED
**Vitiligo and melasma**						
**Polypodium leucotomos**	Indirect inhibition via downregulation of opsin-3 [102]	/	/	Yes, inhibition of Th1 cytokines (IL-2, IFNγ, TNFα), IL-6, increase in IL-10 [128]	/	Increased [103]
**Vitamin C**	Inhibition	/	No effect on melanocyte proliferation at physiological levels [129]	Variable results: downregulation of NFκB, IL-6, TNFα has been described. [130]	Decrease in UV-induced Monocyte Chemoattractant Protein (MCP-1) and CXCL8 [131]	Significantly photoprotective at 10% topically, not photoprotective orally [132]
**Vitamin E**	Inhibition [133]	Inhibition [134]	α-tocopherol decreases UV-induced melanocyte proliferation [134]	Decrease in TNF-alpha [135] suppressing IL-1 and IL-6, COX-2 and CRP [136]	d-α-tocopherol blocks whereas natural d-γ-tocopherol elevates VCAM-1 [137].. Inhibition of MCP-1 and CXCL8 [138]	No or minimal increase [139,140]
Vitiligo						
**Catalase/superoxide dismutase**	Extracellular SOD inhibits UV-induced melanogenesis by tyrosinase inhibtion [141]	/	Extracellular SOD inhibits UV-induced melanocyte proliferation [141]	SOD induces neutrophil apoptosis [142] SOD mimetics inhibit TNF-alpha, IL-1beta, and IL-6 [142]	/	Increased [143]
**Ginkgo biloba**	Inhibition of tyrosinase activity [144]	/	/	Rat cerebral ischemia model: decrease in IL-1, IL-6, p-STAT3, p-JAK2 [105]	Rat cerebral ischemia model: decrease in CXCL10 and CXCL11 [105]	Increased [104]
Melasma						
**Zinc**	Zinc sulphate inhibits tyrosinase [145]		Increased melanocyte proilferation [146]	Inhibition of NFkB [147]	/	/
**Niacinamide**	Dose-dependent inhibition in melanocytes co-cultured with keratinocytes [148]	Inhibition of melanosome transfer [57]	/	Reduction of UV-induced immunosuppression [149]	Downregulation of CCL2 [150]	No change in minimal erythema dose [151]
**Cysteamine**	Inhibition of tyrosinase [152]	/	/		Reduces CCL2 and CCL5 [153]	/
**Sylimarin**	Inhibition of tyrosinase [154]	/	/	Rat liver ischemia model: reduction of Fas/FasL, HMGB1 and CD45 [155] Hepatocellular carcinoma cell line: Upregulation of IL17D and IL17RB, downregulation of IL1A, IL1B, IL2RG, IL6, IL8 [156].	Inhibition of IL-1 induced MCP-1 [157]. Human hepatocellular carcinoma cell line: downregulation of CCL2, CCL20, CXCL1, CXCL2, CXCL3, CXCL6, CXCR4 [156].	Inhibition of UV-induced oxidative stress [158]
**Pycnogenol**	Inhibition of tyrosinase and melanin synthesis [159].	/	/	Modulator of TLR signaling. Induction of IL10 [160].	/	Significant increase in the MED [161].
**Tomato/lycopene**	Inhibition of tyrosinase [162]	/	/	Inhibition of IL-8 and MCP-1 [163]	Adipocytes: decrease of TNFα-mediated induction of IL-6 and MCP-1 [164]. Decrease in IL-1β [164]	Increase in MED [165,166]

Supplementary Table S3: Summary of the placebo-controlled trials using antioxidants for vitiligo and/or melasma.

	Vitiligo			Melasma			
	Sign. better	Non-sign. Better	Not better	Sign. better	Non-sign. Better	Not better	# trials
**Vitamin C**	-	-	-	7	2	-	9
**Catalase/superoxide dismutase (SOD)**	4	1	3	-	-	-	8
**Polypodium leucotomos**	2	1	1	2	-	1	7
**Niacinamide**	-	-	-	1	2	1	4
**Vitamin E**	3	-	1	-	-	-	4
**α-lipoic acid**	1	1	1	-	-	-	3
**Sylimarin**	-	-	-	-	2	1	3
**Cysteamine**	-	-	-	2	-	-	2
**Ginkgo biloba**	1	1	-	-	-	-	2
**Glutatione**	-	-	-	2	-	-	2
**Tomato/lycopene**	-	-	-	2	-	-	2
**Vitamin B12**	-	-	2	-	-	-	2
**Mulberry extract oil**	-	-	-	1	-	-	1
**Rucinol serum**	-	-	-	1	-	-	1
**Pycnogenol**	-	-	-	1	-	-	1
**Selenium**	-	1	-	-	-	-	1
**Tumeric**	1	-	-	-	-	-	1
